# Peripheral endotoxin exposure in mice activates crosstalk between phagocytes in the brain and periphery

**DOI:** 10.21203/rs.3.rs-4478250/v1

**Published:** 2024-06-07

**Authors:** Jake Boles, Oihane Uriarte Huarte, Malú Gámez Tansey

**Affiliations:** University of Florida; University of Florida; University of Florida

**Keywords:** Neuroinflammation, Single-cell RNA sequencing, Central-peripheral crosstalk, Lipopolysaccharide, PIPseq

## Abstract

**Background:**

Inflammation is a central process of many neurological diseases, and a growing number of studies suggest that non-brain-resident immune cells may contribute to this neuroinflammation. However, the unique contributions of specific immune cell subsets to neuroinflammation are presently unknown, and it is unclear how communication between brain-resident and non-resident immune cells underlies peripheral immune cell involvement in neuroinflammation.

**Methods:**

In this study, we employed the well-established model of lipopolysaccharide (LPS)-induced neuroinflammation and captured brain-resident and non-resident immune cells from the brain and its vasculature by magnetically enriching cell suspensions from the non-perfused brain for CD45 + cells. Then, we identified immune subtype-specific neuroinflammatory processes using single-cell genomics and predicted the crosstalk between immune cell subtypes by analyzing the simultaneous expression of ligands and receptors.

**Results:**

We observed a greater abundance of peripheral phagocytes associated with the brain in this model of neuroinflammation, and report that these professional phagocytes activated similar transcriptional profiles to microglia during LPS-induced neuroinflammation. And, we observed that the probable crosstalk between microglia and peripheral phagocytes was activated in this model while homotypic microglial communication was likely to be decreased.

**Conclusions:**

Our novel findings reveal that microglia signaling to non-brain-resident peripheral phagocytes is preferentially triggered by peripheral inflammation, which is associated with brain infiltration of peripheral cells. Overall, our study supports the involvement of peripheral immune cells in neuroinflammation and suggests several possible molecular signaling pathways between microglia and peripheral cells that may facilitate central-peripheral crosstalk during inflammation. Examining these molecular mediators in human disease and other rodent models may reveal novel targets that modify brain health, especially in comorbidities characterized by peripheral inflammation.

## Background

Inflammation is a key component in a growing number of neuropsychiatric and neurodegenerative diseases[1–3], and its role in brain health ranges from protective to injurious. In the brain, microglia are often the first to be implicated in pathogenic neuroinflammation[4] due to their place as brain-resident professional phagocytes and the long-standing dogma that the brain is “immune privileged.” However, the infiltration of peripheral innate and adaptive immune cells has been reported by multiple groups in human diseases as well as rodent models created to study those diseases[5]. For example, evidence for immune dysregulation in cerebrospinal fluid and non-microglial myeloid cell influx in the brain in Alzheimer’s disease (AD) is mounting[6–8], and T-cell autoimmunity has been described in Parkinson’s disease (PD)[9, 10]. Immune cell infiltration has been similarly observed in animal models of Alzheimer’s disease (AD)[11–13], frontotemporal dementia (FTD)[14], and PD[15, 16], and several of these studies have revealed a disease-modifying role of these non-resident immune cells in these models. These observations have led to a revised view of the brain as an immune specialized organ and of neuroinflammation as involving key contributions of non-resident peripheral immune cells[17, 18].

Although the presence of peripheral immune cells at endpoint is well established in brain diseases, an understanding of the relationship between the brain and the peripheral immune system is still nascent. Immune cell traffic across the blood-brain barrier (BBB), especially during neuroinflammation, is well documented[19, 20]. *In vivo* brain imaging studies have demonstrated the ability of both innate[21, 22] and adaptive immune cells[23] to pass through the brain’s endothelium. The brain’s lymphatics system, hosted by the meninges, may also be a source of non-resident immune cells in the brain, although this remains to be experimentally supported. Immune cell surveillance in the brain’s lymphatic spaces appears to be a constitutive phenomenon[18, 24], and cerebrospinal fluid (CSF) immunity appears to influence neuroinflammation[25–27], but migration of immune cells from CSF to parenchyma is not a well-established occurrence. As such, our study focuses on the relationship between the brain and blood-borne immune cells in response to an inflammatory stimulus in the periphery.

The molecular features that underpin peripheral immune cell diapedesis into the brain are still being discovered. A large body of literature describes the features of the endothelium that mediate parenchymal entry by immune cells, including the capture of α-integrins on immune cells by endothelial adhesion molecules and the increased binding of P-selectin on endothelial cells with P-selectin glycoprotein ligand 1 on immune cells during neuroinflammation[24], and modifying these features of the immune-endothelium relationship appear to be therapeutically useful in autoimmune diseases such as multiple sclerosis (MS)[28, 29]. However, the molecular drivers preceding an immune-endothelium interaction offer an additional array of potentially translationally valuable molecules. These drivers are likely to be glial in origin. Indeed, microglial communication with plasma cells via interleukin (IL-) 10 has been demonstrated in brain *Tyrpanosoma brucei* infection[30], and microglial crosstalk with monocytes via C-C chemokine ligand 2 (CCL2) has been shown during brain *Toxoplasma gondii* infection[21]. Advances in single-cell genomics offer much to be learned about the communication between glia and peripheral immune cells. High-throughput sequencing assays with single-cell resolution enable the inference of intercellular communication via the analysis of simultaneously upregulated ligands and their corresponding receptors in putative sender and receiver cells[31], which in turn may allow an unbiased discovery of signaling axes that underlie central-peripheral immune crosstalk.

Additionally, recent single-cell RNA sequencing studies have underscored the heterogeneity that can exist in a tissue[32, 33] and identified cell-specific immune processes during neuroinflammation. One single-nucleus RNA sequencing study profiling the human ventral midbrain defined microglial subtypes enriched in *IL1B* and *GPNMB* expression associated with idiopathic PD[34], and another study defined several microglial subtypes associated with AD that were then validated in independent datasets and with immunohistochemistry[35]. Studies like these have motivated a greater appreciation for microglial heterogeneity, emphasizing the complex multi-dimensionality of microglial functions during development, health, and disease[36]. Similarly, a study using spatial and single-cell transcriptomics in mice demonstrated rich molecular heterogeneity in astrocytes across brain regions and experimental disease states[37]. However, the examination of inflammatory phenotypes during neuroinflammation has been largely limited to microglia and astrocytes, likely due to their overpowering presence over non-resident immune cells in brain-derived datasets.

To examine central-immune crosstalk experimentally, we employed a well-established model of neuroinflammation induced by acute peripheral lipopolysaccharide (LPS) exposure and interrogated the brain with single-cell genomics. We made several methodological decisions to reliably capture peripheral immune cells associated with the brain, which we expected to be rare in number under baseline conditions but notably increased in number during active inflammation. First, we employed the PIPseq platform for single-cell RNA sequencing[38], which enabled the query of up to 20,000 cells per sample. Second, we dissociated the brain into single-cell suspensions and isolated only CD45 + cells from these suspensions. Third, given the critical role of the brain vasculature in enabling peripheral immune cell entry to the brain parenchyma, we omitted transcardiac perfusion prior to brain harvesting. Although this approach limits our ability to definitively conclude that non-resident immune cells captured here are truly parenchymal, it provides a larger and more relevant population of cells with which to interrogate central-peripheral crosstalk, as intercellular communication is likely to be occurring over a distance and across the brain’s borders[39, 40]. The peripheral LPS-induced neuroinflammation model was chosen here due to its rich foundation in the literature, allowing us to anchor and interpret our findings within the context of other published studies. The experimental approach we have chosen here provides support for much of what is known about this model, including the mobilization of monocytes and neutrophils to the brain after acute LPS exposure and the induction of CCL2 as a possible driver of this brain-homing behavior. However, we also reveal many other signaling systems that may underlie peripheral phagocyte extravasation and identify immune processes that are unique to each immune cell type captured here, enabling future studies of these systems to interrogate and validate new health-modifying targets during disease conditions.

## Methods

### Mice

C57BL/6J (Strain #000664) and progranulin-deficient (*Grn*−/−) mice (B6(Cg)-*Grn*^*tm1.1Aidi*^/J, Strain #013175) were obtained from Jackson Laboratories and housed in individually ventilated cages and maintained with *ad libitum* access to standard rodent chow on a 12:12 light-dark cycle in a conventional animal facility in the McKnight Brain Institute vivarium at the University of Florida. All procedures were approved by the University of Florida Institutional Animal Care and Use Committee and followed the *Guide for the Care and Use of Laboratory Animals from* the National Institutes of Health (NIH Publications No. 80 – 23, revised 1996).

To compare immune cell isolation procedures from brain, 4- to 10-month-old C57BL/6J and *Grn−/−* mice were euthanized via rapid decapitation and brains were rapidly extracted. To examine a widely used model of neuroinflammation with our optimized brain immune cell isolation and the PIPseq single-cell genomics platform, 3- to 5-month-old male C57BL/6J received a single intraperitoneal (i.p.) injection of lipopolysaccharide (1.5 × 10^7^ EU/kg) from *Escherichia coli* 0111:B4 (Sigma, #L2630) or an equivalent volume of sterile saline. Twenty-four hours post-injection, mice were euthanized via rapid decapitation and brains were quickly extracted. Critically, brains were processed without perfusion. As described above[19], peripheral immune cells have been observed to enter the brain from the neurovasculature, so we omitted transcardiac perfusion to capture the intercellular communication between microglia and circulating immune cells that may drive this extravasation to identify subtypes of peripheral immune cells that preferentially communicate with microglia during inflammation.

### Brain dissociation protocols

#### Collagenase VIII, DNase I, and Percoll

Brains were extracted from C57BL/6J or *Grn−/−* mice and bisected. One hemisphere was finely minced, digested with 1.4U/mL collagenase VIII (Sigma, #C2139) and 1mg/mL DNase I (Sigma, #DN25) in RPMI 1640 medium (ThermoFisher, #11875119) for 15min at 37°C after which the enzymes were inactivated with the addition of 10% fetal bovine serum (FBS) in RPMI 1640 medium. Digested tissue was manually triturated using fire-polished Pasteur pipettes and filtered through a 70μm nylon cell-strainer, leaving a single-cell suspension. Tissue lysates were suspended in a solution made of 37% Percoll (Sigma, #P1644) in Hank’s balanced salt solution (HBSS−/−; ThermoFisher, #14175103). A layer of 70% Percoll in HBSS−/− was carefully deposited underneath the tissue layer and a layer of 30% Percoll in HBSS−/− was slowly dispensed on top of these two phases. This layered solution was centrifuged for 30 minutes at 400 × *g* with braking disabled to avoid collapsing the layers, after which the top phase containing cellular debris and myelin was discarded. Cells were aspirated from the lower interphase, washed in phosphate buffered saline (PBS), and carried forward to surface staining and flow cytometry.

#### Adult Brain Dissociation Kit and CD45 magnetic separation

The other hemisphere from the above mice was treated with Miltenyi Biotec Adult Brain Dissociation Kit (ABDK, #130-107-677). Hemispheres were cut into 8–12 small pieces and placed into gentleMACS C-tubes (Miltenyi Biotec, #130-093-237) with dissociation solution prepared as instructed by the manufacturer’s protocol. When cells were being isolated for single-cell RNA sequencing, 5μg/mL actinomycin D, 10μM triptolide, and 27.1μg/mL anisomycin or an equal volume of dimethyl sulfoxide (DMSO) as used previously [41] were added to the dissociation solution to enable the evaluation of transcriptional artifacts induced by this method in this sample type. Tissue was subjected to the 37C_ABDK_01 protocol on the gentleMACS Octo Dissociator with heaters (Miltenyi Biotec, #130-096-427). Lysate was filtered through a 70μm nyclon cell-strainer with Dulbecco’s PBS with calcium, magnesium, glucose, and pyruvate (D-PBS). Myelin and cell debris was removed using Debris Removal Solution according to manufacturer’s instructions, which involved resuspending cells in a solution of 0.9mL Debris Removal Solution and 3.1mL D-PBS, gently layering D-PBS above this solution, and centrifuging at 3000 × *g* for 10 minutes at 4°C with slower braking. The top two phases were discarded, cells were washed gently in D-PBS, and red blood cells were lysed with Red Blood Cell Removal Solution diluted 1:10 in distilled water for 10 minutes at 4°C. Lysis was quenched with the addition of 10x volume of D-PBS with 0.5% bovine serum albumin (BSA) added. Cells were resuspended in PBS in the case of a follow-up study or carried to CD45 + cell enrichment (see below).

To enrich for immune cells, cell suspensions were subjected to magnetic separation with antibodies conjugated to magnetic beads. Cells were suspended in 90μL of a buffer made of autoMACS Rinsing Solution (Miltenyi Biotec, #130-091-022) with 0.5% BSA (BB), to which 10μL CD45 MicroBeads (Miltenyi Biotec, #130-052-301) was added. Labeling occurred over 15 min at 4°C. Labeling was quenched with the addition of 20X volume BB. Labeled cells were added to MS columns (Miltenyi Biotec, #130-042-201) in a magnetic OctoMACS Separator (Miltenyi Biotec, #130-042-108). Columns were washed thrice with BB to elute unlabeled cells, which were collected only in the second experiment, and labeled cells were eluted in BB using the provided column plungers. Cells were washed in PBS and subjected to surface labeling and flow cytometry or cell capture and library preparation.

#### Multi-color flow cytometry

To compare tissue dissociation procedures, cells were transferred to a V-bottom 96-well plate (Sigma, #CLS3896–48EA) and centrifuged at 300 × *g* for 5 minutes at 4°C. Pelleted cells were incubated with an antibody cocktail described in Table S1 with LIVE/DEAD Fixable Aqua (Invitrogen, #L34957) for 20 minutes at 4°C. Cells were washed twice in PBS and fixed with eBioscience IC Fixation Buffer (Invitrogen, #00-8222-49) for 30 minutes at 4°C. After one wash, cells were resuspended in 300μL FACS buffer (0.25mM EDTA, 0.01% NaN_3_, and 0.1% BSA in PBS) and analyzed using a BD LSRFortessa. Compensation was achieved using SPHERO Supra Rainbow beads (Spherotech, Inc., #SRCP-35–2A) by setting laser voltages to achieve fluorescent intensity measurements in each channel equal to those in previous experiments. The compensation matrix was created with the acquisition of single-stained AbC Total Antibody Compensation Beads (ThermoFisher, #A10497) for antibodies or ArC Amine Reactive Compensation Beads (ThermoFisher, #A1034) for the LIVE/DEAD dye.

#### Single cell RNA library preparation with PIPseq and next-generation sequencing

Single-cell RNA library preparation was performed using the PIPseq T20 kit (Fluent BioSciences) following the manufacturer’s instructions (revision 7.3). Briefly, 40,000 CD45 + cells per sample were isolated and loaded into PIPs to capture 20,000 cells. Cells were lysed and barcoded mRNA was isolated to perform cDNA synthesis and amplification. cDNA quality was checked using a Qubit dsDNA High Sensitivity assay (ThermoFisher, #Q323854) and High Sensitivity DNA Kit (Agilent, #5067 – 4626) on a Bioanalyzer 2100 (Agilent) before library preparation was performed. Integrity of cDNA libraries were assessed with a High Sensitivity DNA Kit on a Bioanalyzer 2100 before samples were sent for 2×150bp paired-end sequencing on an Illumina NovaSeq 6000 (Illumina) at the Interdisciplinary Center for Biotechnology Research (ICBR) core at University of Florida.

#### Data pre-processing and quality control

FASTQ files were processed through the PIPseeker pipeline v.2.0, which handles barcode and paired-end read matching and trims adapters off read 2 to enable genome alignment. Genome alignment is carried out through PIPseeker using the STAR aligner[42] and the GRCmm39 mouse reference genome (from GENCODE). The resulting BAM file is parsed and transcripts are counted to generate a dataset containing all present gene barcodes with their unique molecular indices, which is then converted to a raw count matrix that contains the counts of each unique molecule in each cell. After PIPseeker processing, all analysis was performed in R v.4.2 and v.4.3, and code needed to reproduce results and visualizations can be found at https://github.com/jakesboles/Boles_et-al_brain_immune_scRNAseq.

The feature-barcode matrix was processed with Seurat v.4[43, 44] in R v.4.2 and v.4.3. Seurat objects were created for each sample and merged. Mitochondrial gene abundance and cell complexity (log_10_ transformed number of counts divided by the log_10_ transformed number of unique genes) were calculated for each cell. Cells with 1) less than 5% mitochondrial genes; 2) at least 250 but less than 6500 unique genes expressed; 3) at least 800 but less than 45000 total gene counts; and 4) at least a cell complexity ratio of 0.8 were carried forward. Cutoff selection was determined through iterative selection and visual examination of relevant quality control metrics.

To handle potential batch effects due to staggered cell capture days and enable cell type identification from different conditions, the dataset was integrated using reciprocal principal component analysis (RPCA). The dataset was separated by cell capture day, and each subset was normalized using SCTransform (v.2) with *method = “glmGamPoi”*[45]. A set of integration anchors was computed, and RPCA was performed with this anchor set. The first 100 principal components (PCs) were computed and the first 30 were carried forward. Dimensional reduction was achieved with the uniform manifold approximation and projection (UMAP) technique with *RunUMAP* and its default settings through Seurat. Nearest neighbors were computed based on the PCA and cell clusters were identified using the Louvain algorithm with *resolution* = 0.2 to allow for coarse clustering and initial cell annotation.

#### Cluster annotation and data cleaning

Coarsely clustered cells were annotated using Seurat’s *F* ∈ *dAllMarkers* function and its default settings, which uses a Wilcox test to determine differentially expressed genes in each cluster compared to all other clusters. These gene lists were interrogated using CellMarker 2.0[46] to infer cell annotations which were confirmed with the localization of canonical marker genes. Further inspection revealed microglial markers (e.g., *P2ry12, Hexb*) present in non-microglia clusters. These clusters were inspected individually with re-integration and sub-clustering. In some cases, a distinct group of cells could be observed that expressed genes typical to that sub-cluster but also to microglia, so these cells were regarded as doublets and removed from the dataset. After this cleaning, the dataset was reintegrated as described above, re-clustered with a low resolution, markers were generated again, and clusters were re-annotated to ensure accurate labeling.

At this point, our dataset consisted of microglia, monocytes/macrophages, B-cells, a cluster consisting of T-cells and natural killer cells, neutrophils, and several non-immune clusters. Each of these groups was run through the chooseR pipeline[47], which performs iterative subsampling and clustering to pick the most robust clustering strategy. The microglial dataset was randomly down-sampled to leave a dataset one-eighth in size for this pipeline due to its computational intensity. The clustering resolutions that yielded the greatest median silhouette score were selected, and markers were identified using Seurat’s *F* ∈ *dAllMarkers* with default settings. Silhouette scores were computed for each subcluster and visualized. Cell identities were designated based on these marker lists, which were applied to the full dataset. After this process, the dataset was pruned of any doublets, reassembled, and re-integrated.

#### Cell proportion analysis

To evaluate whether LPS induces a change in the cellular composition captured by our pipeline, the Speckle package[48] was used, which transforms population proportions, fits a linear model for each cell type with the predictors of interest, and estimates *p*-values with an empirical Bayes shrinkage of variances using the Limma package[49]. We transformed the proportions in our dataset with the arcsine transformation, included only *in vivo* treatment (LPS or saline) as a predictor, and present the FDR-adjusted *p*-values for each cell type. Although groups of cell types are presented separately, all 20 populations were passed through one analysis, and the *p*-values are adjusted accordingly.

#### Transcriptional artifact analysis

The annotated dataset was divided into subsets for analysis of artifactual signature induction. Due to the infrequency of certain cell types, basophils, mast cells, and neutrophils were merged into one granulocyte dataset, αβ T-cells and yδ T-cells were merged into one T-cell dataset, and plasma cells and B-cells were merged into one B-lymphocyte dataset. All non-immune cells were merged into one CD45-cell dataset. All other populations were subset individually. A cross entropy test[50] was conducted in each dataset to determine whether the UMAP embeddings of inhibitor-treated cells and vehicle-treated cells differed significantly from one another, and the Kullback-Leibler divergences and Holm-adjusted *p-*values are shown for each comparison. Artificial activation modules were taken from differential expression analyses from pseudobulked single-cell datasets in ref.[41] and added to the cell cluster objects with Seurat’s *AddMod**e**Sc* or e function. Artificial gene module induction was visualized using scCustomize’s (https://cran.r-project.org/web/packages/scCustomize/index.html) wrapper functions for Seurat’s visualization tools. To separate artificially activated populations, cells were clustered and artifact module scores of each cluster were plotted using scCustomize’s *V ln.Plot*_*s*_*cCus → m* function and the contribution of each experimental group to each cluster was visualized with the *dio Bar Plot* function from the dittoSeq package[51], revealing subsets of cells that displayed higher expression of the artifactual gene module and were dominated by samples that did not receive inhibitors during dissociation. Artificially activated populations were removed from the full dataset before downstream analysis as were non-immune cells.

#### Intercellular communication analysis

Differences in intercellular communication between brain immune cells evoked by peripheral endotoxin were inferred and analyzed using two complementary R packages. CellChat[52] uses mass action models and differential expression analyses to infer cell-specific signaling patterns within experimental groups of interest. The law of mass action based on the average expression of a ligand in one cell type and a receptor in another cell type is used to infer the probability of communication, and the significance of this interaction is determined by whether this probability is higher than that amongst randomly permuted cell groups. MultiNicheNetR[53] uses the differential state analysis as described by the muscat R package[54], which includes cell-level mixed models and statistical tests on aggregated pseudo-bulked data. This package makes sample-level inferences on intercellular communication states, allowing us to examine both the extent and variability of the differential expression of ligand-receptor pairs.

#### Pseudo-bulked differential expression analysis

For each immune cell subset, the effect of LPS on gene expression was assessed, identifying groups of differentially expressed genes (DEGs) for each cell type. Raw gene counts from each cell in each sample were aggregated using Seurat’s *Aggregate Expression* to create a pseudo-bulked dataset, and immune cell subtypes were separated for DEG analysis. Differential expression was done with DESeq2[55]. A gene was designated to be differentially expressed if its absolute value of the log2 transform of the fold change with LPS exceeded 2 and its Benjamini-Hochberg adjusted *p*-value was less than 0.05.

#### Gene set enrichment analysis (GSEA)

A GSEA[56] was performed in immune cell types using the ClusterProfiler package[57]. Specifically, ranked gene lists were passed through ClusterProfiler’s *compareClusters* function, where Gene Ontology knowledgebase’s[58, 59] Biological Process pathways with greater than 30 genes and fewer than 300 genes were used as the gene sets of interest. Gene ranking came from DESeq2’s effect size shrinkage procedure using the *apeglm* algorithm[60]. Enrichment scores calculated during the random walk were normalized based on gene set and overlap size, and p-values were adjusted using the Benjamini-Hochberg correction. To compare the enrichment of biological pathways between immune cell types during LPS exposure, representative terms from the top 30 pathways with the lowest corrected *p*-value were selected from each cell type.

#### AD and PD gene list curation

To examine the cell-specific expression of risk genes associated with neurodegenerative disease, gene lists were assembled from genome wide association studies (GWAS) and meta-GWAS. A set of 31 genes associated with AD was used as in ref.[61] and ref.[62]. For PD, the 62 unique eQTL-nominated genes from ref.[63] were sorted on the meta-*p*-value that included random effects. Both gene lists were converted from human symbols to mouse symbols, leaving 30 orthologs for AD and 59 orthologs for PD.

#### Statistical analysis

All statistical analyses were performed in R v4.2–4.3. When evaluating the efficacy of different brain dissociation techniques, data were analyzed with a two-way mixed ANOVA using the Afex package (https://cran.r-project.org/web/packages/afex/index.html), with genotype as a between-subjects predictor and protocol as a within-subjects predictor. All pairwise comparisons were made using the Emmeans package (https://cran.r-project.org/web/packages/emmeans/index.html) with Tukey’s correction, and compact letter displays (CLD) were generated with the Multcomp package (https://cran.r-project.org/web/packages/multcomp/index.html). All pairwise comparisons were made within each fraction with Tukey’s correction and CLDs were generated. The Geisser-Greenhouse correction was used when a departure from sphericity was observed in our data according to the Performance package’s (https://cran.r-project.org/web/packages/performance/index.html) *check*_*s*_*phericity*, which uses Mauchley’s test. All other statistical analyses performed within our single cell RNA sequencing analysis are described above. Tables displaying ANOVA and cross entropy test results were generated using the GT package (https://gt.rstudio.com/).

## Results

### CD45 magnetic separation followed by PIPseq captures the complexity of the immune microenvironment in the mouse brain

**Figure 1:**
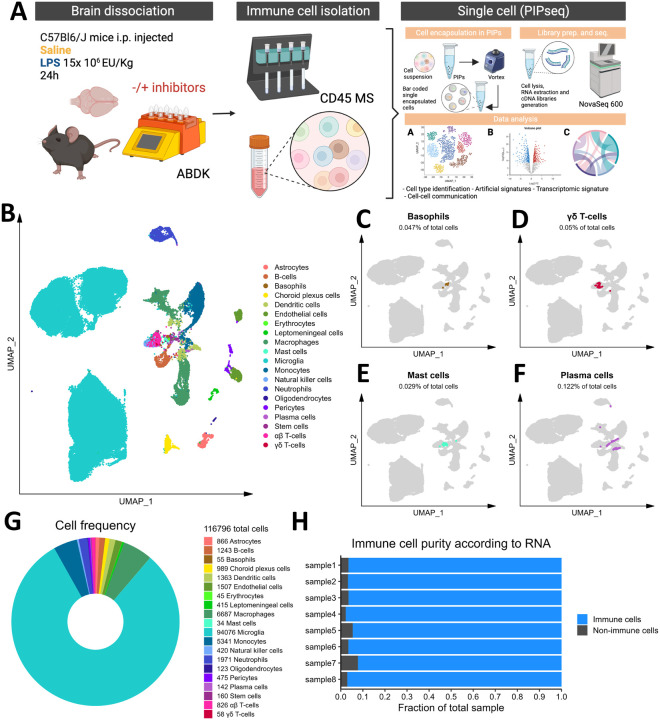
Microfluidics-free single-cell RNA sequencing in brain immune cells. A) Experimental workflow, describing *in vivo* and *ex vivo* treatments and data processing workflow. B) Cellular populations identified, backed by the rationale provided in Figures S2-S8. Basophils (C), mast cells (D), plasma cells (E), and γδ T-cells (F) are highlighted as rare subsets. G) Total cell counts, counts of individual populations, and donut plot showing relative frequencies of our dataset. H) Stacked bar chart for each sample showing the relative frequencies of immune cells and non-immune cells.

To interrogate central-peripheral immune crosstalk during neuroinflammation, we employed the well-established systemic endotoxin-induced neuroinflammation model by injecting C57BL/6J mice with LPS (1.5 × 10^7^ EU/kg, i.p.) or an equivalent volume of sterile saline, and immune cells were isolated from brain tissue with ABDK and CD45 MS 24 hours post-injection. This brain dissociation strategy was selected over a strategy involving collagenase VIII digestion and Percoll-mediated selection of immune cells, which we and many others have used previously[13, 14], based on a preliminary experiment we performed that revealed higher yield and better purity of immune cells with the ABDK and CD45 MS (**Figure S1**). Since the use of enzymes to dissociate brain tissue has been shown to artificially induce immune signatures, mainly in microglia[41, 64], we included inhibitors of transcription, translation, and cell division or an equivalent volume of DMSO vehicle during dissociation as described previously[41] to examine the extent to which microglia and nearby immune cells are affected by this dissociation method. After isolation, 40,000 cells per sample were input to capture 20,000 cells in particle-templated instant partitions (PIPs), which were then lysed for downstream cDNA library preparation and sequencing[38] (Fig. 1A).

Initial graph-based clustering revealed 25 populations of cells, which were divided into six broad populations consisting of B-cells, CD45-cells, microglia, myeloid antigen presenting cells (APCs), neutrophils, and T- and natural killer cells (NKs), based on differential expression testing and the expression of canonical markers such as *Ptprc, Itgam, Ngp, Cd3e, Ms4a1*, and *Nkg7*(**Figure S2**). We examined each cluster in detail with an iterative subsampling procedure and differential expression testing to confidently annotate cells (**Figure S3** to **Figure S8**). The “B-cell” population was grouped into 6 subtypes (**Figure S3**) that each expressed B-cell markers such as *Cd79a, Ms4a1*, and *Iglc3* (**Figure S3D-F**), although one cluster also expressed T-cell genes such as *Cd3e* and *Nkg7* and was designated as a cluster of doublets and removed (**Figure S3G-I**). The CD45-population was grouped into 17 subtypes which expressed markers for astrocytes (e.g. *Aqp4, Gja1, S100b)*, choroid plexus cells (e.g. *Folrl)*, endothelial cells (e.g. *Cldn5)*, leptomeningeal cells (e.g. *Col1a2, Dcn, Slc38a2)*, oligodendrocytes (e.g. *Olig1)*, and pericytes (e.g. *Acta2*) (**Figure S4**). The microglial population expressed classic microglial markers such as *Aif1, C1qc, Hexb, P2ry12*, and *Tmem119* (**Figure S5**). The myeloid APCs were clustered into 18 subtypes (**Figure S6A-C**). Several cell types were identified based on marker gene expression, including macrophages (expressing *Mrc1, Cd163, Ms4a7)*, monocytes (expressing *Ccr2, Ly6c2, Arg1)*, dendritic cells (expressing *Itgax, Cd83, Cd86)*, plasma cells (expressing *Igkc, Vpreb3)*, and erythrocytes (expressing *Hba-a1*) were identified (**Figure S6D-P**). The neutrophil population consisted of 10 subtypes expressing neutrophil markers such as *Mmp8, Ngp, S100a8*, or *S100a9* (**Figure S7A-G**). The microglial marker *P2ry12* was expressed in one small cluster, which were classified as doublets and removed (**Figure S7H-I**). Lastly, the T- and NK cell population was grouped into11 subtypes which were annotated as αβ T-cells (expressing CD3 alleles, *Cd8b1)*, NK cells (expressing *Gzma* and *Klrb1c)*, γδ T-cells (expressing γ- and δ- T-cell receptor chains), erythrocytes (expressing *Hba-a1)*, macrophages (expressing *Marco)*, mast cells (expressing *Mcpt4)*, basophils (expressing *Plac8* and *Cebpa)*, and stem cells (expressing many immature and proliferating cell markers (**Figure S8**).

In summary, we captured 20 different cell types from the mouse brain and its vessels that consisted of immune cells, including microglia, monocytes, macrophages, B- and T- cells, NKs, dendritic cells, and neutrophils, as well as a some non-immune and/or brain-resident cells (Fig. 1B). We also identified rare immune populations including basophils, mast cells, plasma cells, and γδ T-cells(Fig. 1C–F). Our cleaned dataset contained over 116,000 cells from only 8 mouse brains, over 90% of which were immune cell populations (Fig. 1G–H), demonstrating the efficacy of this CD45 MS-based approach in enriching the mouse brain and neurovasculature for immune cells.

### Myeloid cells are vulnerable to an artificial activation state due to enzymatic dissociation

**Figure 2:**
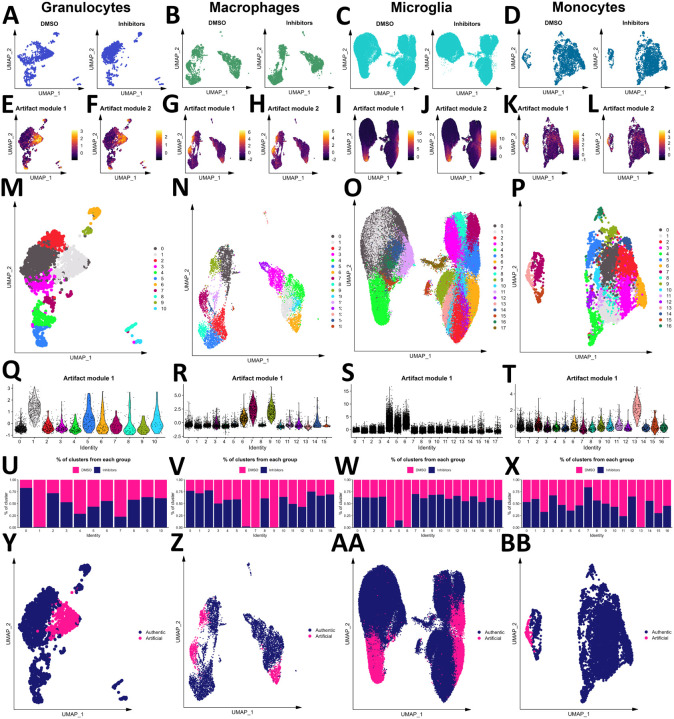
Myeloid cells are vulnerable to enzyme-induced artifactual activation. The embeddings of granulocytes (A), macrophages (B), microglia (C), and monocytes (D) from samples treated with and without inhibitors. Scores of two artifactual gene modules from the literature are shown for granulocytes (E-F), macrophages (G-H), microglia (I-J), and monocytes (K-L). Granulocytes (M), macrophages (N), microglia (O), and monocytes (P) were clustered, and the first artifact module scores were plotted by cluster for each population (Q-T, respectively). The contribution of samples treated with or without inhibitors during dissociation to each granulocyte (U), macrophage (V), microglia (W), and monocyte (X) subset. Final designation of artifact status for granulocytes (Y), macrophages (Z), microglia (AA), and monocytes (BB).

Enzymes to dissociate the mouse brain have been shown to induce artificial gene activation[41, 64]. Hence, we examined to what extent this already defined artificial microglia subpopulation was induced by the ABDK method and whether nearby circulating immune cells were affected. To determine whether the embeddings of cells differed based on inhibitor exposure, we performed a cross-entropy test[50] on immune cell subsets and the non-immune cell subset. This analysis revealed that granulocytes (composed of the neutrophil, basophil, and mast cell populations), macrophages, microglia, and monocytes were affected by enzymatic dissociation (**Table 1**). The low-dimensional embeddings of each of these populations in the inhibitor-treated and vehicle-treated samples are shown in Fig. 2A–D. The up-regulation of two published artifactual gene modules[41] can be seen in cells not treated with inhibitors during dissociation (Fig. 2E–L). To separate those artifactually activated cells, these three populations were clustered (Fig. 2M–P), artifact module expression was examined by cluster (Fig. 2Q–T), and the contribution of each experimental group to each cluster was calculated (Fig. 2U–X), revealing one cluster of granulocytes, three clusters of macrophages, three clusters of microglia, and one cluster of monocytes that were designated as artifactually activated (Fig. 2Y–BB). These clusters were removed ahead of biological interrogation of the LPS model.

### Systemic administration of endotoxin activates central-peripheral immune crosstalk

**Figure 3:**
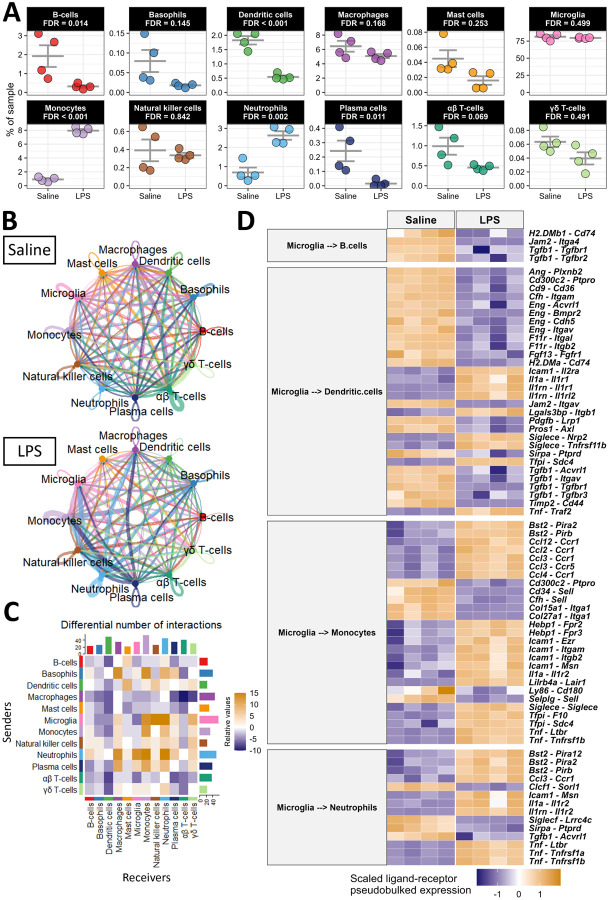
Peripheral LPS exposure activates dynamic central-peripheral immune crosstalk. A) Population frequencies of immune cells from Figure 2 with FDR-adjusted p-values from comparing the frequencies in saline- and LPS-treated samples shown for each. B) Circle plot showing the information flow between cells inferred by CellChat for each condition. Lines and bubbles are colored by sender cell type. C) Heatmap showing the differential number of interactions between all combinations of sender (y-axis) and receiver (x-axis) cells. Bars on the top and right show the column-wise and row-wise, respectively, sum of cells. D) Heatmap showing the top 75 ligand-receptor pairs from MultiNicheNet, when microglia were the senders of interest and B-cells, dendritic cells, monocytes, and neutrophils were the receivers of interest. The heatmap is colored by the z-scored pseudobulked expression of the ligand-receptor pairs, and each column is a sample.

To begin dissecting the unique contributions of peripheral immune cells during LPS-induced neuroinflammation, we evaluated whether intraperitoneally administered LPS induces a change in the cellular composition of our brain immune-cell samples. We observed an increase in neutrophils and monocytes and a decrease in B-cells, dendritic cells, and plasma cells in/near the brain with LPS (Fig. 3A). The relative abundance of other immune cells and the non-immune cells captured here were not significantly affected by LPS (Fig. 3A, **Figure S9**). To determine the reason for these changes in immune cell composition with LPS, we inferred the communication of cells using multiple R packages, including CellChat[52] and MultiNicheNetR[53]. The overall strength of cellular communication was weakened by LPS (**Figure S10A-B**), and the nature of this cellular communication was modulated by LPS (**Figure S10C**), suggesting that circulating endotoxin alters immune cell communication between brain and blood. Indeed, the inferred communication between specific pairs of cells is modulated by endotoxin exposure (Fig. 3B–C). For example, macrophages were expressing fewer ligands with predicted receptors while neutrophils were expressing more ligands (Fig. 3C). Interestingly, microglia were expressing more ligands whose predicted receptors were expressed by peripheral immune cells, especially monocytes, NKs, and neutrophils, with LPS (Fig. 3C).

We hypothesized that microglia, the brain-resident cells in our dataset, would primarily govern the traffic of cells to and from the brain. We used MultiNicheNetR to predict the specific ligands expressed by microglia and the specific receptors expressed by those immune cells that are differentially associated with the brain after peripheral LPS exposure. This analysis revealed that chemoattractant and adhesion signals from microglia to B-cells and dendritic cells are largely down-regulated during peripheral LPS exposure. Specifically, several integrins, including *Itgam, Itgav*, and *Itgb2* were downregulated in dendritic cells as were their putative binding partners *Eng, F11r*, and *Cfh* in microglia (Fig. 3D). Communication from microglia to dendritic cells and B-cells through transforming growth factor β (TGFβ) was disrupted by peripheral LPS exposure, and the adhesion of these cells to microglia through major histocompatibility complex class II (MHC-II) receptors (e.g., *H2-DMa, H2-DMb1*, and *Cd74*) and junctional adhesion molecules (JAMs; especially *Jam2*) was also decreased with systemic LPS exposure (Fig. 3D). Additionally, we inferred an elevation of several chemoattractant and adhesion signals from microglia to monocytes and neutrophils. The C-C chemokine, interleukin (IL)-1, and tumor necrosis factor (TNF) systems were inferred to be highly active between microglia and these two peripheral myeloid populations with peripheral LPS exposure, potentially creating a driving force for the migration of these cells. Additionally, the expression of adhesion molecules, especially *Icam1* and *Bst2*, were upregulated in microglia as were their binding partners in monocytes and neutrophils (Fig. 3D). This analysis revealed novel potential binding partners that suggest an extravasation of mononuclear cells in the brain, including the interaction between tetherin (encoded by *Bst2*) and several paired-Ig-like receptors (*Pira2, Pira12*, and *Pirb*), orthologous to human leukocyte immunoglobulin-like receptors, and the interaction between heme binding protein 1 (*Hebp1*) and several formyl peptide receptors (*Fpr2* and *Fpr3)*. CellChat analysis corroborates the cell-specific modulation of signaling classes during peripheral LPS exposure. CellChat inferred a loss of incoming JAM and TGFβ and other adhesion molecules in dendritic cells and B-cells during peripheral LPS exposure, while monocytes and neutrophils were predicted to be receiving more interleukins, adhesion ligands, and chemokines (**Figure S10D**). In agreement with the above results, microglia were again predicted to be sending less TGFβ, more C-C chemokines, more TNF, and different adhesion factors (**Figure S10E**).

**Figure 4:**
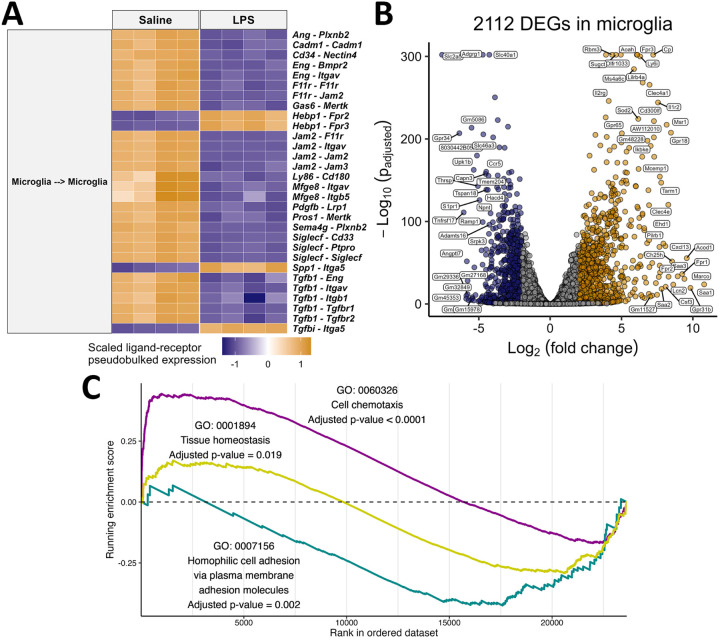
Microglial adhesion and homeostasis support is disrupted by peripheral inflammation induced by intraperitoneally-administered lipopolysaccharide. A) The top 30 ligand-receptor pairs between microglia inferred from MultiNicheNet are shown on the right y-axis. The heatmap is colored by the z-scored pseudobulked expression of the ligand-receptor pairs, and each column is a sample. B) Volcano plot showing the change in expression of genes in pseudobulked microglial samples. Differentially expressed genes are defined as having adjusted *p* < 0.05 and absolute log2 fold change > 2. C) Random walk from a gene set enrichment analysis on selected Gene Ontology pathways in genes ordered based on differential expression in microglia.

Finally, we examined the potential disruption of communication between microglial cells inferred by CellChat (Fig. 3C) using MultiNicheNetR. Of the top 30 differentially regulated ligand-receptor pairs, 26 were downregulated during peripheral LPS exposure, affirming that microglia-microglia crosstalk was disrupted by peripheral inflammation (Fig. 4A). Specifically, peripheral LPS exposure induced a loss of TGFβ signaling, integrin binding, and adhesion molecule expression between microglia. From the inferred loss of adhesion molecules, we hypothesized that LPS-exposed microglia would be more chemotactic. In support of this, a GSEA revealed that homophilic cell adhesion in microglia was disrupted by LPS and cell chemotaxis was strongly enriched in LPS-treated microglia (Fig. 4B–C). In addition, we hypothesized that in response to peripheral inflammation, microglia would be less homeostatic due to the overall downregulation of *Tgfb1* and its receptors, as observed previously. A large gene set associated with tissue homeostasis was enriched in control microglia, in support of this hypothesis (Fig. 4B–C). Overall, peripheral LPS exposure disrupted the inferred signaling between microglia, antigen presenting cells, and other microglia. Instead, microglia expressed ligands for receptors highly expressed in peripheral myeloid cells to a greater extent, which may explain the greater frequency with which monocytes and neutrophils were found near the brain in our dataset.

### Peripheral inflammation induces shared and cell-type specific transcriptomic programs in the brain

**Figure 5:**
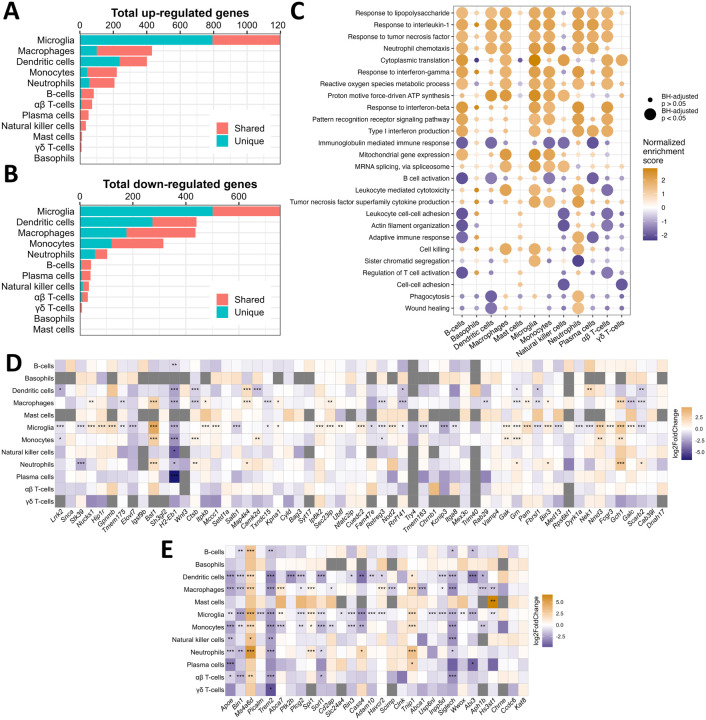
Unique and shared transcriptomic programs in all immune cells dissociated from brain in response to peripheral endotoxin exposure. A) Bar chart showing the total number of genes with adjusted *p* < 0.05 and log_**2**_ fold change > 2 on pseudobulked datasets. Bar colors show the number of DEGs that are shared by at least one other cell type or unique to that cell type. B) Same as A but showing genes that have log_**2**_ fold change < −2. C) Dot plot showing the enrichment scores from select GO: BP pathways in all immune cell types after pseudobulked differential expression analysis and gene ranking. Dots are sized according to whether the enrichment met statistical significance (adjusted *p* < 0.05) and colored according to the normalized enrichment score. D) Heatmap showing the log_**2**_ fold change in expression from saline to LPS of the mouse orthologs of genes associated with the heritability of PD. The x-axis is ranked by ascending *p*-value from ref. [63]. E) Heatmap showing the log_**2**_ fold change in expression from saline to LPS of the mouse orthologs of genes associated with the heritability of AD. The x-axis is ranked by ascending *p*-value. This list is borrowed from ref. [61] and ref. [62]. *p < 0.05, **p < 0.01, ***p < 0.001

As the mobilization and communication of immune cells in the peripheral LPS exposure model is complex, we aimed to distinguish how different cell types contribute to peripheral LPS-induced neuroinflammation. We performed differential expression and gene set enrichment analyses in each cell type by creating pseudo-bulked datasets at the sample level and employing standard bulk RNA sequencing tools. Microglia displayed the greatest number of differentially expressed genes (DEGs), while adaptive immune cells and small granulocyte populations showed few DEGs, and peripheral phagocytes regulated a middling number of DEGs (Fig. 5A–B). We illustrate how many of these DEGs were unique to their respective cell type or shared by at least one other, showing a range of transcriptomic overlap depending on cell type (Fig. 5A–B).

To infer the functional state of each cell type, we employed the well-established gene set enrichment analysis (GSEA)[56, 57] on genes ranked by their differential expression due to peripheral LPS exposure. Several gene sets implicated in LPS signaling, cytokine response, protein synthesis, and metabolic activation were enriched in multiple cell types, suggesting that many immune populations, both in and near the brain, were metabolically and immunologically responsive to peripheral LPS exposure (Fig. 5C). However, gene sets associated with cytotoxicity, including the production of TNF, were enriched only in macrophages, monocytes, microglia, and neutrophils, while gene sets associated with cellular adhesion, cytoskeletal organization, and adaptive immune activation were disrupted in B-cells, T-cells, and NKs (Fig. 5C). Interestingly, neutrophils upregulated genes associated with phagocytosis and wound healing, while microglia and other peripheral phagocytes downregulated or showed no change in these pathways (Fig. 5C). These data suggest that the neuroimmune profile induced by peripheral LPS exposure is a product of the activity of many distinct immune cells, and certain inflammatory processes within this profile may be dominated by the activity of peripheral immune cells.

Finally, we curated lists of risk factors identified for Parkinson’s disease (PD) and Alzheimer’s disease (AD) in meta-genome wide association studies (meta-GWAS)[61 –63] and examined the change in expression of these genes in each cell type due to an inflammatory stimulus. Microglia displayed regulation of many of these genes in response to peripheral LPS exposure, and some genes are uniquely regulated by microglia, including *Gpnmb, Tmem163, Picalm*, and *Slc2a4*(Fig. 5D–E). However, many of these genes were also differentially regulated by peripheral myeloid cells in response to peripheral LPS exposure, including *Bst1, Gch1, Lrrk2, Satb1, Apoe, Plcg2, Siglech, Sorl1, Spi1* (Fig. 5D–E). Most immune populations captured in this study up-regulated *Ms4a6d*and *Tnip1* and downregulated *H2-Eb1, Bin1*, and *Trem2* after peripheral LPS exposure (Fig. 5D–E). Interestingly, certain genes differed in the direction of their regulation between microglia and peripheral immune cells in response to peripheral LPS exposure, including *Galc, Nek1, Abca7*, and *Cass4*, while only peripheral populations regulated genes such as *Camk2d, Ctsb, Grn, Map4k4, Abca1, Aph1b, Cd2ap*, and *Ptk2b*. Overall, our findings reveal the existence of immune cell subset-specific expression of genes associated with risk for neurodegeneration in response to peripheral LPS, consistent with the growing perspective that genes that confer risk to neurodegenerative disease are linked to the peripheral immune system.

## Discussion

In this study, we aimed to profile brain-immune interactions in an unbiased manner, capitalizing on recent advances in single-cell RNA sequencing and bioinformatics to assess immune cell-specific responses to peripheral inflammation induced by acute endotoxin exposure and infer the crosstalk between distinct immune cell populations. Peripheral immune cell activation is well established in the peripheral LPS model, enabling us to anchor our findings from this high-throughput single-cell analysis in robust literature. Additionally, the diapedesis of immune cells into the brain is an increasingly postulated feature of human disease, so we deliberately profiled the mouse brain without clearing its blood to capture this dynamic process and its molecular underpinnings.

First, we replicated previous findings that enzymatic dissociation can induce false gene signatures in microglia[41, 64], which must be considered with care when interrogating the brain’s immune response to stimuli. The artifactual gene signature, comprised mostly of immediate-early genes, heat shock protein genes, and some chemokines and cytokines[41], was well preserved in our study. However, in addition to microglia, we noted the induction of this false signature in other myeloid cells, including neutrophils, macrophages, and monocytes, making the proper control over this false signature even more critical. Importantly, the pharmacological inhibition of this genomic artifact established by previous studies[41] was also well preserved in our study, thereby enabling an artifact-free query into brain-immune interactions.

In the peripheral LPS model, we observed the trafficking of neutrophils to the brain, which is a documented effect of peripheral endotoxin administration. This movement of neutrophils due to peripheral LPS exposure has been confirmed by others using immunohistochemical approaches[65] and flow cytometry techniques[66, 67]. *In vivo* two-photon imaging has revealed physical contact between microglia and neutrophils in the brain of mice treated with peripheral LPS[22], consistent with the heightened communication between microglia and neutrophils we report here, especially the increased expression of adhesion molecules, such as *Icam1* and *Msn* respectively, on these two cell types. We also demonstrated the increased expression of IL-1 receptors, TNF receptors, and C-C chemokine receptors on neutrophils, which have been shown to induce the migration of neutrophils[68–70] and are likely enabling downstream adhesion interactions with microglia.

The migration of monocytes towards the brain due to peripheral LPS, another established consequence of the model and other models of peripheral inflammation, was also observed here. Fate mapping with reporter-mouse models[71] and flow cytometry[72] have demonstrated the infiltration of monocytes to the brain during LPS exposure and the necessity of CCL2 in mediating this traffic. Our single-cell data support the induction of *Ccl2* signaling from microglia to monocytes after peripheral LPS exposure, but our analysis suggests that several other microglial C-C chemokine ligands, including CCL3 and CCL4, and monocyte C-C chemokine receptors, including CCR1 and CCR5, may also be involved. Monocytic infiltration of the brain has been reported in a model of hepatic inflammation, where TNF was implicated as a key regulator of this traffic[73]. Our intercellular communication analyses support the role of TNF, and in recent work we observed increased monocytes in the brain of animals that experienced colonic inflammation induced by dextran sulfate sodium[74]. Moreover, monocytes have been implicated as key disease effectors in models of central nervous system damage including models of ischemia, neurodegeneration, and demyelination[75], suggesting that the relationship we report here between brain resident and peripheral phagocytes has broad disease implications. Additionally, physical contact between microglia and infiltrating monocytes triggered by peripheral inflammation has not been reported, but the induction of adhesion systems between these cell types we report warrants further exploration of this relationship and its consequences on brain health.

Importantly, our scRNAseq approach reveals the unique and overlapping contributions of different cell types to the broad effects of peripheral inflammation that have been reported previously. Peripheral LPS exposure induces interferon signaling in the brain which is generally attributed to microglia[76], but our single-cell approach suggests that interferon production and signaling is induced in both innate and adaptive immune cells near the brain, in line with other reports from non-CNS and *ex vivo* systems[77–80]. TGFβ signaling between brain cells is increased during aging and ischemia[81] which is likely a neuroprotective mechanism[82]. It is known that LPS antagonizes TGFβ in microglia[83], but we report herein that microglial TGFβ signaling to peripheral immune cells is also disrupted after peripheral LPS exposure, suggesting that peripheral inflammation dysregulates homeostatic immune crosstalk. Leukocyte adhesion, which we found is increased between microglia and neutrophils via *Bst2* and *Icam1* and microglia and monocytes via *Icam1, Bst2*, and several integrins, but dysregulated in NKs and adaptive immune cells, has not been directly examined in the brain under conditions of peripheral inflammation, but it has been observed in the human lymphatic system[84], *in vitro* tumor systems[85, 86], and mouse kidney[87] after LPS exposure. Finally, we suggest that several non-microglial immune cell types contribute to the classic readouts of peripheral LPS-induced neuroinflammation, including TNF production, interleukin production, and metabolic activation[88], a finding that has implications for future cell type-specific targeting in therapeutic interventions.

Further, the mobilization of disease-related genes in the brain in response to peripheral inflammation shows complex cell-type specificity. Specifically, we found that genes implicated in the heritability of PD and AD were regulated by microglia during peripheral inflammation, supporting the growing evidence that the pathogenesis of these diseases is linked to immune function[89–92]. However, many of these genes were regulated by peripheral immune cells, either in addition to or differently from microglia, suggesting that more intense focus needs to be placed on cells outside the brain to obtain an accurate picture of how brain health is compromised by chronic systemic inflammatory conditions. This perspective is beginning to be adopted by the field with respect to genes implicated in the endolysosomal system including leucine-rich repeat kinase (LRRK2)[93], progranulin (GRN), and glycoprotein non-metastatic B (GPNMB) as risk genes for several neurodegenerative diseases[3, 14, 94]. Interestingly, disease-related genes were regulated largely by myeloid cells in our study. Yet, it is known that genomic programs characterized by the same genes may be activated in T-cells during aging[62] and vascular cells during AD[61], suggesting that the cell-type specificity of risk-gene expression we report here may be more complex in the chronically inflamed brain or disease states.

It might be said that a clear limitation of the presented study is our omission of transcardiac perfusion prior to immune cell isolation and single-cell transcriptomics. As described above, this methodological approach was intentional due to the growing evidence that immune cells enter and communicate with the brain from the circulation[24], yet we acknowledge that it poses some restrictions on the interpretation of the data. For example, we report a simultaneous upregulation of adhesion molecules in microglia and peripheral phagocytes, suggesting that these cells are making contact. While adhesion between neutrophils and microglia during neuroinflammation has been reported[22], we cannot determine whether neutrophils and monocytes are contacting microglia, the endothelium prior to diapedesis, or another non-resident cell type in the brain in this study. Also, although we can detect rarer immune populations, including several adaptive immune cell subsets and small granulocyte subsets, and measure their differential expression in the peripheral LPS model, we do not know whether these cells are parenchymal or associated with the vasculature and the extent to which their reactivity in this model is influential to brain health. Yet, we believe that other studies adopting the strategy presented here can identify the molecular foundation of T-cell entry into the brain, for example, after observing that entry with orthogonal measures. In general, we believe the merit of this approach lies in the unbiased discovery of molecules that may drive the involvement of non-microglial immunity in brain diseases which can then be validated experimentally.

Due to the CD45 MS used to enhance the immune cell purity of our samples, we captured only small amounts of astrocytes, pericytes, and endothelial cells in our scRNAseq dataset, which limits the insight we can gain about the role of these cells during peripheral LPS-induced neuroinflammation. The neurovascular unit, comprised of these cells, is a key intermediary in central-peripheral immune crosstalk. LPS may be injurious to the blood brain barrier (BBB) in certain doses and conditions[95], and leukocytes adhere to the endothelium before entering the brain parenchyma[96]. In our study, we cannot discern whether monocytes and neutrophils are migrating to the brain due to increased cellular adhesion with endothelial cells or to BBB damage. We also cannot determine the extent to which microglial chemoattractant signals are reaching these peripheral myeloid cells directly or astrocytes and endothelial cells, which are known producers of chemoattractants and interleukins[97, 98], are mediating this crosstalk. Yet, unraveling the complexity of this multicellular system is a tractable proposition by using FACS or MS procedures to isolate both immune and neurovascular cells coupled with the scalability offered by PIPseq[38].

## CONCLUSIONS

In summary, we have demonstrated the utility of an accessible and robust platform to delineate the unique contributions of brain-resident and brain-associated immune cells to peripheral inflammation-induced brain inflammation. Our single-cell genomics approach suggests that microglia preferentially communicate with peripheral myeloid cells rather than other microglia after acute peripheral LPS exposure, which may have far-reaching consequences for maintaining brain health[99]. The nature of this central-peripheral immune crosstalk in other animal models of neuroinflammation represents a source of untapped insight for human disease that will be feasible to interrogate using a methodology similar to that employed here. Whether microglia are mobilizing the adaptive immune system under conditions of acute versus chronic peripheral inflammation directly or indirectly through long-term communication with peripheral antigen-presenting cells are critically important unanswered questions with clear therapeutic implications[9, 100]. Finally, whether innate immune cells are recruited to the brain in a similar manner in other cases of acute inflammation is also of interest to the field, as is the extent to which abrogation of this recruitment is beneficial for brain health. These lines of study, enabled by the experimental approach described herein, may unlock new targets to improve brain health through modulation of central-peripheral neuroimmune crosstalk.

## Data Availability

Raw single-cell RNA sequencing data are accessible through NCBI’s Gene Expression Omnibus via the GEO Series accession number GSE245309 (https://www.ncbi.nlm.nih.gov/geo/query/acc.cgi?acc=GSE245309). All code needed to reproduce the analysis and figures in this manuscript can be found at https://github.com/jakesboles/Boles_et-al_brain_immune_scRNAseq.

## Abbreviations

ABDK: Adult Brain Dissociation Kit
AD: Alzheimer’s disease
APC: antigen-presenting cell
BB: beads buffer
BBB: blood-brain barrier
BSA: bovine serum albumin
CCL: C-C chemokine ligand
CD45 MS: CD45 magnetic separation
CSF: cerebrospinal fluid
DEG: differentially expressed gene
DMSO: dimethyl sulfoxide
D-PBS: Dulbecco’s PBS
eQTL: expression quantitative trait loci
FACS: fluorescence-activated cell sorting
FBS: fetal bovine serum
FDR: false discovery rate
FTD: frontotemporal dementia
GPNMB: glycoprotein non-metastatic B
GRN: progranulin
GSEA: gene set enrichment analysis
GWAS: genome-wide association study
HBSS: Hank’s balanced salt solution
IL: interleukin-
JAM: junctional adhesion molecule
LPS: lipopolysaccharide
LRRK2: leucine-rich repeat kinase 2
MHC-II: major histocompatibility complex class II
MS: multiple sclerosis
NK: natural killer cell
PBS: phosphate-buffered saline
PC: principal component
PD: Parkinson’s disease
RPCA: reciprocal principal component analysis
scRNAseq: single-cell RNA sequencing
TGFβ: transforming growth factorβ
TNF: tumor necrosis factor
UMAP: uniform manifold approximation and projection
